# The Influence of Abiotic Stress Factors on the Morphophysiological and Phytochemical Aspects of the Acclimation of the Plant *Rhodiola semenowii* Boriss

**DOI:** 10.3390/plants10061196

**Published:** 2021-06-11

**Authors:** Nina V. Terletskaya, Nazym K. Korbozova, Nataliya O. Kudrina, Tatyana N. Kobylina, Meruert S. Kurmanbayeva, Nataliya D. Meduntseva, Tatyana G. Tolstikova

**Affiliations:** 1 Department of Biodiversity and Biological Resources, Faculty of Biology and Biotechnology, Al-Farabi Kazakh National University, Al-Farabi av., 71, Almaty 050040, Kazakhstan; naz-ik@mail.ru (N.K.K.); meruyert.kurmanbayeva@kaznu.kz (M.S.K.); nat.mdnt@gmail.com (N.D.M.); 2 Institute of Genetic and Physiology, Al-Farabi av., 93, Almaty 050040, Kazakhstan; kobylina.tatyana.n@mail.ru; 3 N.N. Vorozhtsov Novosibirsk, Institute of Organic Chemistry, Siberian Branch of Russian Academy of Science, 630090 Siberia, Russia; tg_tolstikova@mail.ru

**Keywords:** water deficit, cold stress, water content, photosynthesis, anatomy, secondary metabolites

## Abstract

Plants of the *Crassulaceae* family are natural accumulators of many medicinal secondary metabolites (SM). This article describes the study of morphophysiological, anatomic and phytochemical responses of immature plants of *Rhodiolla semenovii* under water deficit and (or) cold-stress conditions. Changes in biomass production due to water content in plant tissues such as a decrease in water deficit and an increase in cold stress were revealed. A significant decrease in the efficiency of the photosynthetic apparatus under stress conditions was noted, based on the parameters quantum efficiency of Photosystem II and electron transport rate and energy dissipated in Photosystem II. The greatest decrease in efficiency was pointed out in conditions of water shortage. The anatomical modulations of root and shoot of *R. semenovii* under stress conditions were found. For the first time, a detailed study of the chemical composition of the ethanol extract of root and shoot of *R. semenovii* under stress was carried out using gas chromatography–mass spectrometry. The qualitative and quantitative composition of SM associated with acclimation to the effects of abiotic stresses was determined. Both nonspecific and specific phytochemical changes caused by the action of water deficiency and cold treatment were identified. It has been shown that the antioxidant system in plant tissues is complex, multicomponent, depending on a number of natural and climatic factors. Further research should be focused on the use of abiotic stressors for the targeted synthesis of bioactive SMs valuable for pharmaceutical use.

## 1. Introduction

Nowadays dry and arid regions cover a vast territory. According to the Netherlands Environmental Assessment Agency [1], they make up about half of the earth’s environment worldwide. Identification of the mechanisms of acclimation of plants to the habitat and resistance to water and temperature stress acquires particular importance in conjunction with the expected further global climate changes.

In addition to the agricultural aspect, the problem of resistance to abiotic stresses is of great natural and ecological importance, since the ability of plants to adapt to specific conditions in different parts of the planet is one of the factors that determine the distribution areas of wild species and the possibility of their introduction [2].

The changes caused by adverse environmental influences, including geo climatic and seasonal changes, external conditions of temperature, humidity, etc., negatively affect many metabolic processes in plants. Alteration of photosynthesis is one of the earliest processes to be affected by negative stress, long before stress-induced changes in plants become visible. For example, temperature fluctuations, a decrease in the availability of nutrients and water affect the parameters of chlorophyll (Chl) fluorescence, CO_2_ assimilation, and a decrease in stomatal conductance and transpiration [3,4]. Among other things, abiotic stresses affect biomass production, often due to water content in plant tissues and PSM (plant secondary metabolism). Likewise, qualitatively and/or quantitatively on SM (secondary metabolites), which perform various functions important for physiological developmental processes, and the content of which can also change during growth and development in response to environmental changes [5,6]. Therefore, there is data that medicinal plants, the vegetation of which takes place against the background of exposure to abiotic stresses, usually exhibit significantly higher concentrations of SM than identical plants of the same species grown in favorable conditions. Perhaps, plant SMs which are synthesized in response to environmental stresses partly determine the ability of plants to survive and adapt to abiotic stress factors [7]. However, so far there is very little information about this well-known phenomenon [8].

There is no doubt that any plant, even those referred to by people as “medicinal”, synthesizes its biologically active substances, first “for itself”. In this way, it can be assumed that, depending on the nature of the stress effect (its intensity, duration, rate of exposure), biologically active structures or compounds allow the plant to flexibly and adequately respond to abiotic stress factors. In this case, the type and concentration of secondary metabolites produced under stress conditions can be determined by the genotype, specific features of physiology, and the stage of plant development. This also suggests that the production of secondary metabolites may be an indicator of a protective response [9,10,11,12].

Therefore, the protective role of plant secondary metabolism (PSM) in oxidative stress was established [13,14,15,16]. It was reported that the widely studied phenylpropanoids, in addition to the formation of structural components in plants (for example, the synthesis of lignin necessary for the formation of the cell wall), also participate in plant defense responses to abiotic stresses [17,18]. An increase in fatty acid unsaturation in the composition of membrane lipids in hypothermia was noted [19]. It was shown that seasonal climatic fluctuations influenced the production of sesquiterpenes, lactones, and phenols. The correlation of the content of these substances in plants with the amount of precipitation and temperature changes was established [20]. It was found that the effect of drought on plants promotes a higher production of such classes of secondary metabolites as terpenes, complex phenols and alkaloids [21,22,23]. The development and understanding of the contribution of individual stress-protective systems to the constitutive and induced resistance of plants was developed. In particular, the information on the role of such classical stress protectors as sugars [24] and proline [25] has expanded significantly in recent years. However, the formation of harmonious ideas about the functioning of plant protective systems under abiotic stresses is complicated. On the one hand, there is a variety of effects depending on the intensity and duration of exposure and the physiological state of plants at the time of exposure. On the other hand, the formation is compounded by a significant dependence of the nature of defense reactions on species characteristics.

The processes of protective stress reactions stimulate metabolic changes that can lead to the biosynthesis of biologically active compounds with pharmaceutical or nutritional value. For centuries, people in one way or another have been using the physiological acclimations of medicinal herbs as a source of improving the biosynthesis of useful bioactive compounds [13,26,27]. Nowadays, the physiology of stress associated with PSM is attracting more attention of researchers and more literary sources indicate the stabilizing role of metabolites [16,28,29,30,31]. Impulses are outlined for new practical approaches to improving product quality through the deliberate application of stress exposure during the cultivation of medicinal plants [8].

Therefore, the changes cause great interest in the synthesis of SM induced by stressful conditions. It causes the interest both from the point of view of understanding the biological processes of aclimation of a plant organism to unfavorable environmental conditions, and from the point of view of the introduction of wild valuable species and the development of pathways for the directed synthesis of plant biologically active substances valuable for pharmacy.

Plants of the *Crassulaceae* family are natural accumulators of many medicinal SM. *R. semenovii* (Regel and Herder) Boriss is a taproot, short-rhizome perennial that grows in moist, rocky soils and along riverbanks in the alpine belt up to 3500 m above sea level, preferring a sunny location. The studies carried out by phytochemists and pharmacologists show that plants of the genus *Rhodiola* and representatives of *R. semenovii*, contain proanthocyanidins, coumarins, flavone glycosides, and organic acids, tannins of the pyrogallic and pyrocatechol groups [32,33]. However, the metabolic profile of *R. semenovii* and possible changes in its metabolites, including drug-active and stress-resistant components, have not been illustrated in any way under different vegetation conditions yet.

The aim of this article was an experimental study of sudden water shortage or cold exposure on immature plants *R. semenovii*. The outcome measures will help to determine the effect of these stressors on changes in the physiological state and content of the main classes of secondary metabolites in the root and shoot of this member of the *Crassulaceae* family. Moreover, it will be useful both for understanding the mechanisms of defense against adverse conditions and for approaches to the targeted synthesis of valuable secondary metabolites.

## 2. Results

### 2.1. Morphophysiological Reactions of R. semenowii Immature Plants under Stress Conditions

**Figure 1:**
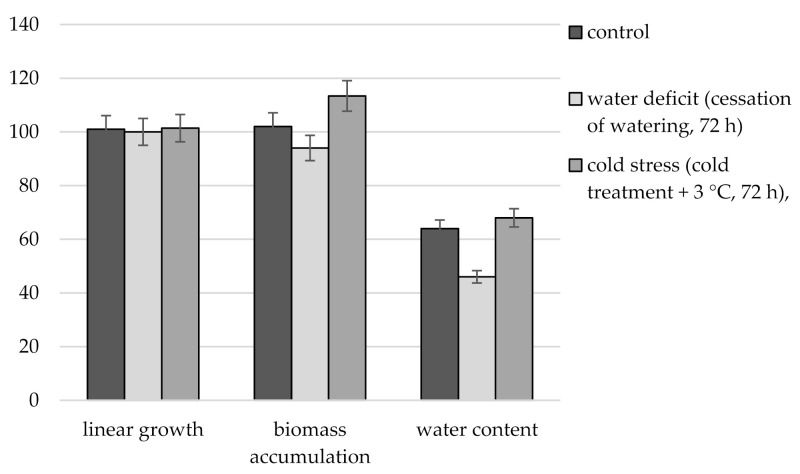
Changes in growth parameters, biomass accumulation and water content of *R. semenowii* under stress conditions.

Comparison of immature *R. semenowii* plants under stress and control conditions showed the absence of linear growth in stressed plants compared to control ones (Figure 1). At the same time, under conditions of water deficit, a significant decrease in biomass was noted, while under conditions of cold stress, its increase. The water content in the tissues of *R. semenowii* under stress conditions of water deficit was low and amounted to 46%, compared to 64% in the control plants. An opposite tendency to an increase in tissue hydration was observed under cold stress, the water content was 68%.

**Figure 2:**
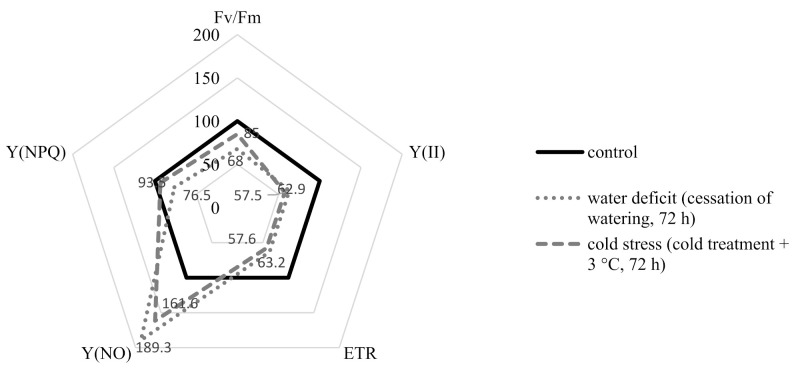
Changes in the activity of the photosynthetic apparatus of *R. semenowii* under stress conditions.

The data presented in Figure 2 show that the stress of *R. semenowii* plants caused by both water deficiency and exposure to cold could be accompanied by a significant decrease in the efficiency of the photosynthetic apparatus. A decrease in the values of the maximum quantum yield of photosystem II (PSII) (ratio Fv/Fm) is shown. The rate of noncyclic electron transport through PSII (ETR) decreased. The values of the parameter of the quantum yield of the unregulated dissipation energy in PSII-Y(NO) increased. However, according to the level of decrease in the quantum yield of regulated energy dissipated in PSII Y(NPQ) and the value of the Fv/Fm index, as well as by the level of increase in Y(NO), damage to the photosynthetic apparatus was less under conditions of cold stress than under conditions of water deficiency.

### 2.2. Changes in the Aspects of the Anatomical Structure of R. semenowii under Stress Conditions

**Figure 3:**
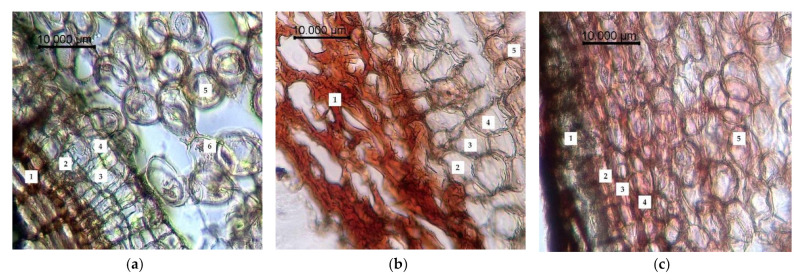
Changes in the anatomical structure of the root of *R. semenowii* under stress conditions: (**a**) control, (**b**) water deficit (cessation of watering, 72 h), (**c**) cold stress (cold treatment +3 °C, 72 h). 1—periderm; 2—fellam; 3—phellogen; 4—phelloderm; 5—parenchyma of the primary cortex; 6—starch grains; scale bar = 10 µm.

**Figure 4:**
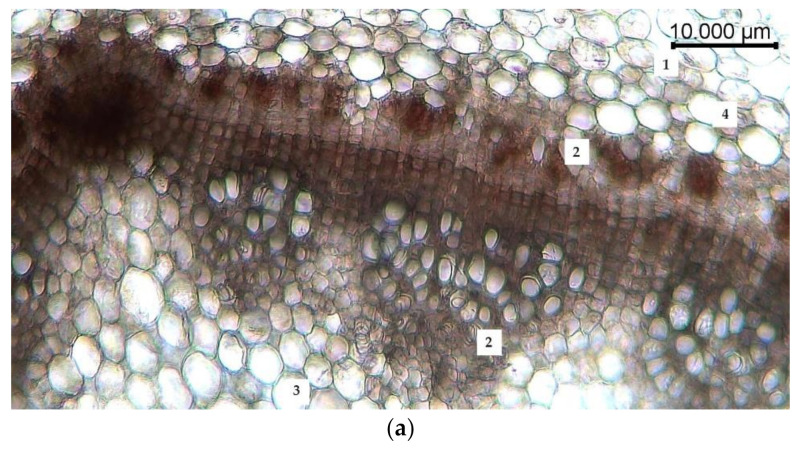

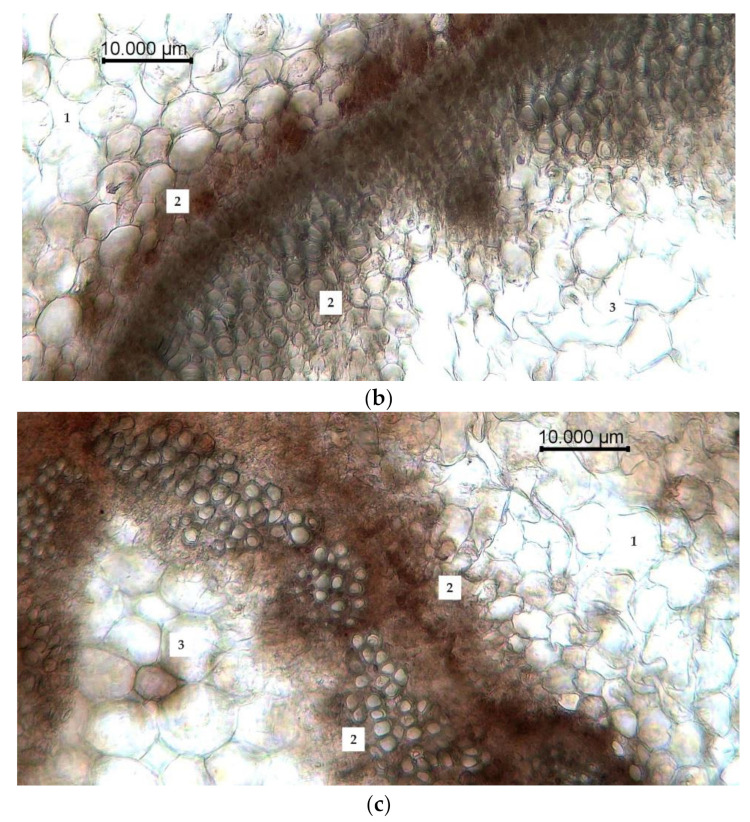
Changes in the anatomical structure of the stem of *R. semenowii* under stress conditions: (**a**) control, (**b**) water deficit (cessation of watering, 72 h), (**c**) cold stress (cold treatment +3 °C, 72 h). 1—assimilation parenchyma; 2—conductive beam; 3—parenchymal cells of the core; scale bar = 10 µm.

**Figure 5:**
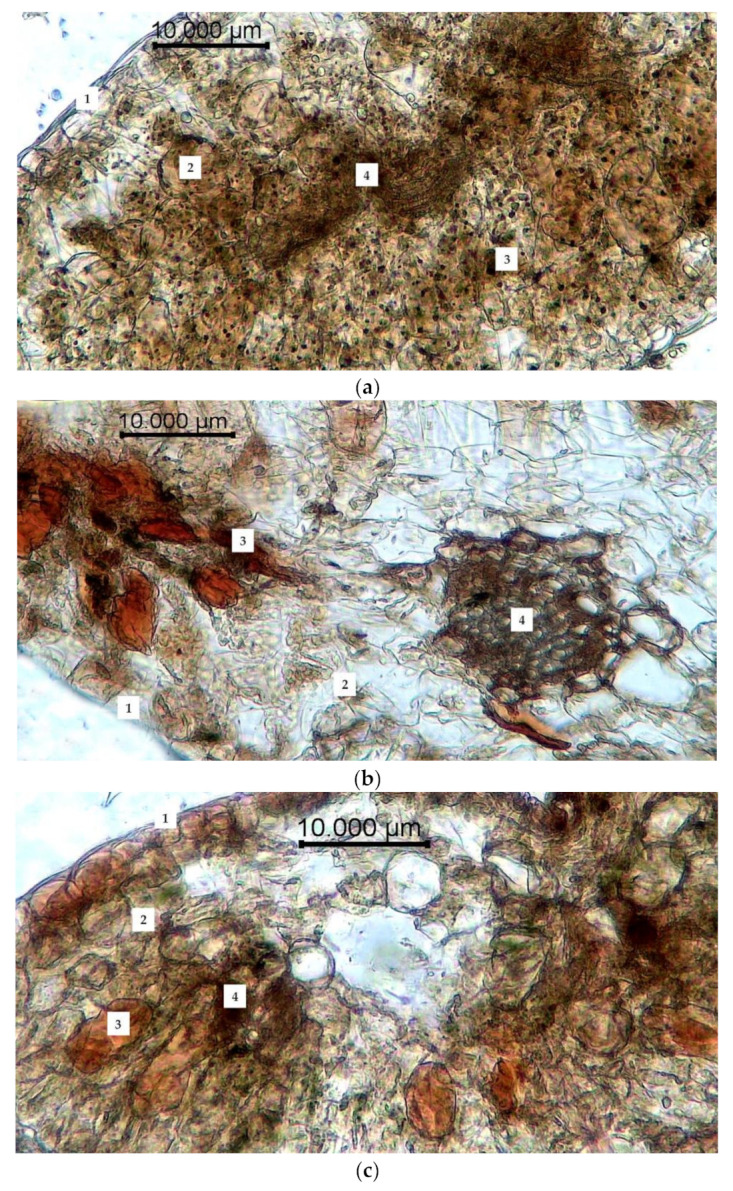
Changes in the anatomical structure of the leaf of *R. semenowii* under stress conditions: (**a**) control, (**b**) water deficit (cessation of watering, 72 h), (**c**) cold stress (cold treatment +3 °C, 72 h). 1—epidermis; 2—mesophyll, 3—inclusions; 4—conductive bundle; scale bar = 10 µm.

The results of anatomical and histological examination are presented in Figure 3, Figure 4 and Figure 5. It was determined that the rhizome of *R. semenowii* had a sparse cell structure in the control plots (Figure 3a). The cross section shows a three-layer periderm. The cell walls of the periderm are covered with suberin and are colored brown. There are three layers such as fellam, phellogen and phelloderm under the periderm. Moreover, there is a loose parenchyma with large cells with numerous small round and oval starch grains and unformed inclusions under the integumentary tissues. The cells of the parenchyma have a rounded-oblong shape.

The conditions of water deficiency led to a significant deformation of the peridermal cells, their flattening and multiple ruptures. Turgor of parenchymal cells is reduced; starch grains are hydrolyzed, and the dye penetrates into cells (Figure 3b).

The cells become more hydrated under conditions of cold stress. In addition, the cells of the primary cortex acquire a more rounded shape, the periderm become denser and they are intensely stained (Figure 3c).

The anatomical study of the stem of *R. semenowii* noted features such as the cells of the epidermis are located in one row; the epiderm is characterized by the presence of a slightly thickened cuticle of the outer wall. The cells of the assimilation parenchyma, located in several rows under the layer of the epiderm, have a rounded-elongated shape and alternate with large intercellular spaces. Single inclusions are found among the cells of the primary cortex. The central cylinder is a conductive system of adjacent conductive bundles arranged in a circle. The pith consists of round or oval shaped parenchymal cells (Figure 4a).

Water deficiency led to changes in the cells of the primary cortex, which became multifaceted, flattened and stretched towards the central cylinder. Conducting bundles were “squeezed” by parenchymal cells, deformed, and destroyed. There were no inclusions. Turgor of parenchymal cells was reduced, and their deformation was noted (Figure 4b).

Inclusions of the primary cortex were displaced to the periphery of the stem and were located in the cells of the epiderm under conditions of cold stress. The cells of the parenchyma increased in size, becoming more rounded and more hydrated, the intercellular spaces were absent (Figure 4c).

The epidermal cells of the *R. semenowii* leaf are arranged in one row and have an oval shape, from the outside they are covered with a cuticle. Cells of the spongy type, the length of which decreases represent mesophyll from the outer to the inner layers. Palisade cells form 2–3 layers. Numerous point inclusions, as well as very large areas of biologically active compounds, were noted in leaf tissues. In the central part of the leaf, there are small collateral-type vascular bundles (Figure 5a).

The water deficiency conditions lead to a change in the shape of cells. Plasmolysis of cells develops; parenchymal cells flatten, stretch and shift towards the conducting beam. Conducting beams are deformed and disintegrated. Biologically active substances are concentrated in the middle of the leaf blade. The stomata plunge into the leaf mesophyll. Cells of the palisade parenchyma are destroyed (Figure 5b).

The leaf blade had acquired sparse cell structure under conditions of cold stress. Hydrated parenchymal cells were increased in size; being located in the central part of the leaf blade they took sharper outlines and the areas of accumulation of biologically active substances were more clearly distinguished (Figure 5c). Wilt and chlorosis of the leaf blades was observed when exposed to both water deficiency and cold stress.

### 2.3. Changes in Plant Secondary Metabolism of R. semenowii under Stress Conditions

GC-MS analysis of *R. semenowii* organs (shoots and roots) growing on control and stress backgrounds revealed the presence of up to 34 compounds (phytochemicals) in each of the studied variants, which can contribute to the medicinal qualities of the plant (Supplementary Table S1). The identification of phytochemicals was confirmed based on peak area, retention time and molecular formula. Analysis of mass spectra showed that stress conditions significantly alter the dominant spectrum of PSM shoots and roots of *R. semenowii* (Supplementary Table S2).

Analysis of the biologically active substances found in *R. semenowii* plants according to the classes of chemical compounds made it possible to reveal certain patterns.

**Figure 6:**
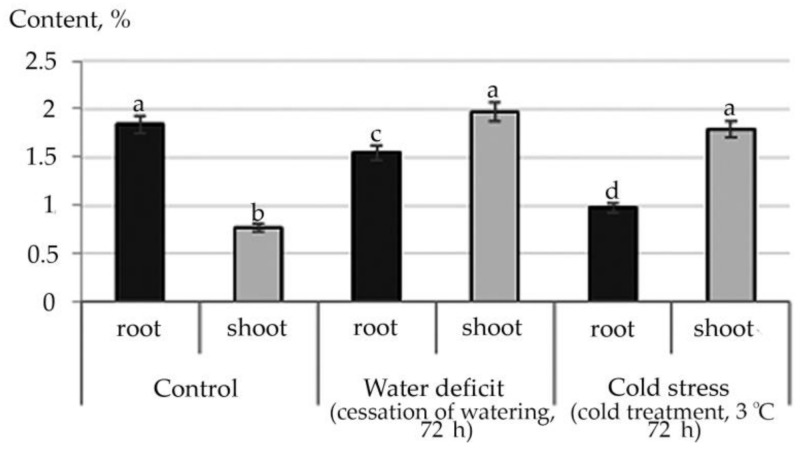
Change in the content of γ-Tocopherol in *R. semenowii* under stress conditions. Values presented are means (±SD). Different letters above the bars represent significant differences at *p* ≤ 0.05, *n* = 3 plants in each of 3 replicates for all treatments.

Therefore, according to the data of the structural group composition in the ethanol extract of the root and stem of *R. semenowii* against stressful backgrounds, a change in the content of substances from the group of ubiquinones was illustrated. An increase in the shoot content of the γ-Tocopherol vitamin under stressful conditions and a decrease in its concentration in the root were noted (Figure 6). At the same time, an increase in the content in shoot 4,8,12,16-Tetramethylheptadecan-4-olide (Supplementary Tables S1 and S2) was shown against the stress background.

Variations in the phytosterols content, exhibiting high biological activity in various physiological processes, were observed. (Supplementary Tables S1 and S2). An increase in the content of β-Sitosterol in root during cold stress was shown, the formation of in root against stressful backgrounds γ-Sitosterol with a higher content during cold treatment than with water deficiency.

The fatty acids were represented by 17-Octadecynoic acid and Propanoic acid, 3-(acetylthio)-2-methyl-; the first one was identified in shoot under control conditions, and the second in root under water deficit conditions (Supplementary Tables S1 and S2).

**Figure 7:**
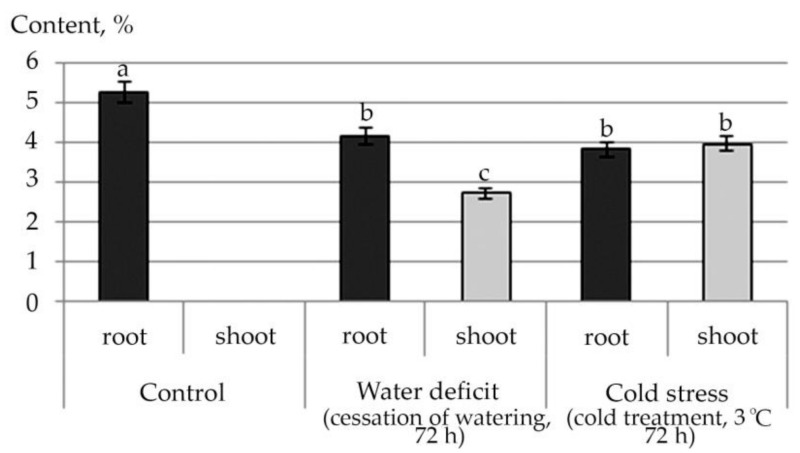
Changes in the content of fatty acid esters in *R. semenowii* under stress conditions by the example of Ethyl 9,12,15-octadecatrienoate (Ethyl 9α-linolenate, linolenic acid ethyl ester). Values presented are means (±SD). Different letters above the bars represent significant differences at *p* ≤ 0.05, *n* = 3 plants in each of 3 replicates for all treatments.

An increase in the content of all detected fatty acid esters in shoot under the conditions of the studied abiotic stresses, and a decrease in almost all of them, except for Ethyl oleate under water stress conditions in root, were noted (Supplementary Tables S1 and S2). This trend is illustrated by the example of linolenic acid ethyl ester, a plant metabolite with antioxidant activity (Figure 7).

**Figure 8:**
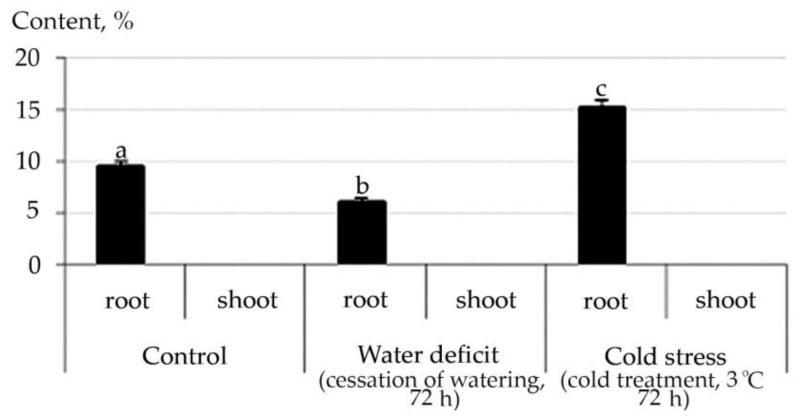
Change in the content of Tetracosyl acetate in root of *R. semenowii* under stress conditions. Values presented are means (±SD). Different letters above the bars represent significant differences at *p* ≤ 0.05, *n* = 3 plants in each of 3 replicates for all treatments.

The pharmacological action of biologically active substances *R. semenowii* is also determined by the content in their composition of aldehydes, glycosides, alcohols, hydrocarbons, amino acids and their derivatives, which have a rather complex structure, the content of which in plant tissues also changed under the influence of stress factors. For example, the content of Tetracosyl acetate (Wax monoesters) was not detected experimentally in shoot of *R. semenowii*. However, in root, we noted a significant change in the content of Tetracosyl acetate under stress conditions: a decrease under water deficit and an increase under cold stress (Figure 8).

A decrease in Cyclopropyl carbinol in root under cold stress, an increase in the content of 1-Docosanol acetate under cold stress, and Cyclopropyl carbinol in root under osmotic stress were demonstrated (Supplementary Tables S1 and S2).

A change in the content of cyclic five-membered ketones, lactones and their derivatives under stress conditions was revealed (Supplementary Tables S1 and S2).

**Figure 9:**
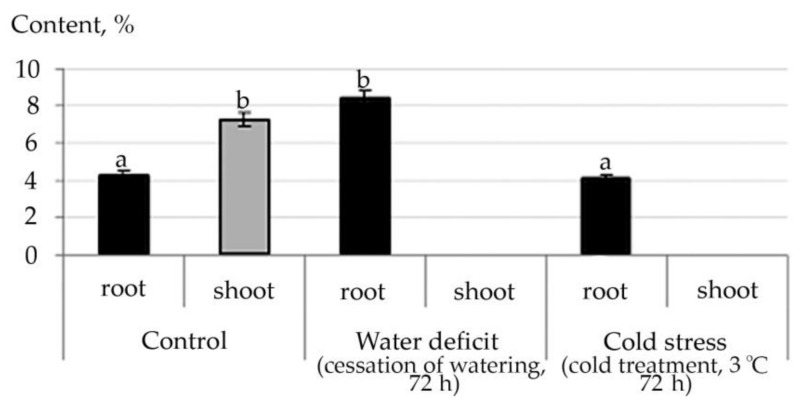
Change in the content of 2-Hydroxy-gamma-butyrolactone in *R. semenowii* under stress conditions. Values presented are means (±SD). Different letters above the bars represent significant differences at *p* ≤ 0.05, *n* = 3 plants in each of 3 replicates for all treatments.

The tendency to an increase in the content of ketones in root was noted under water deficiency, while cold stress caused a decrease in the content of such detected ketones as 4-Cyclopentene-1,3-dione (shoot), 2-Propanone, 1-(acetyloxy)-(root), 2-Cyclopenten-1-one, 2-hydroxy-(root and shoot), 1,2-Cyclopentanedione, 3-methyl-(root and shoot) (Supplementary Tables S1 and S2). An increase in the concentration of psychoactive oxybutyrate 2-Hydroxy-gamma-butyrolactone in root under water deficit conditions and a decrease in it under conditions of cold stress with complete absence in stress conditions in shoot were revealed (Figure 9).

**Figure 10:**
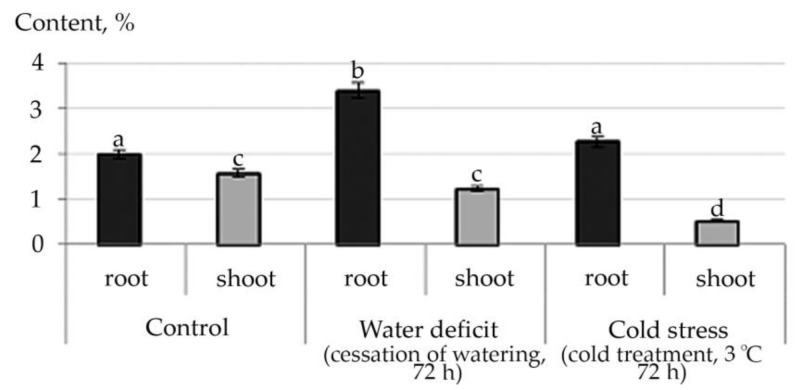
Changes in the content of furan derivatives in root and in shoot using the example of 2 (5H)-Furanone in *R. semenowii* under stress conditions. Values presented are means (±SD). Different letters above the bars represent significant differences at *p* ≤ 0.05, *n* = 3 plants in each of 3 replicates for all treatments.

Furan and Pyran derivatives containing active alcohol, aldehyde and ketone groups, which also exhibit high biological activity, are of particular importance in the formation of the direction and specificity of the pharmacological action of *R. semenowii* preparations (Supplementary Tables S1 and S2, Figure 10).

Phenols were presented only in shoot under control conditions and in the root against the background of water deficiency (Supplementary Tables S1 and S2).

The ethanol extract of *R. semenowii* contains derivatives (esters) of Benzoic acid (aromatic carboxylic acid). At the same time, an increase in the concentration of Benzoic acid, pentadecyl ester under water and cold stresses in root and disappearance in shoot were revealed, while the content of Benzoic acid, tridecyl ester under conditions of both osmotic and cold stress increased in both shoot and root. The content of Benzoic acid, heptyl ester, increased under conditions of cold stress in root and was not detected in shoot, while under conditions of water deficit it tended to decrease in all the studied organs. However, under conditions of water deficit, neoplasms of Benzoic acid, tetradecyl ester in root were noted (Supplementary Tables S1 and S2).

**Figure 11:**
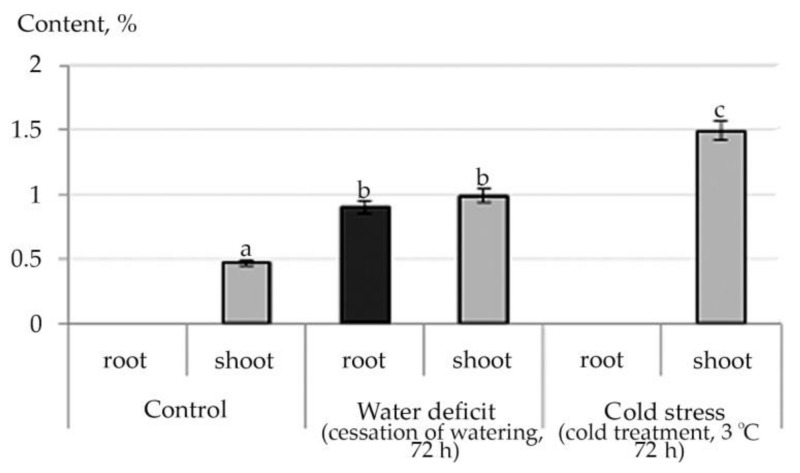
Change in the content of Hexadecanoic acid, 1-(hydroxymethyl)-1,2-ethanediyl ester in *R. semenowii* under stress conditions. Values presented are means (±SD). Different letters above the bars represent significant differences at *p* ≤ 0.05, *n* = 3 plants in each of 3 replicates for all treatments.

The change in the content of esters of saturated monobasic acids by the example of an ester of palmitic (hexadecanoic) acid is illustrated in Figure 11.

A significant increase in the content of Hexadecanoic acid, 1- (hydroxymethyl) -1,2-ethanediyl ester under in shoot stresses and the appearance under conditions of water deficit in root was revealed. The appearance under the influence of osmotic stress of Formic acid, 2,6-dimethoxyphenyl ester in shoot (Supplementary Tables S1 and S2) was shown.

**Figure 12:**
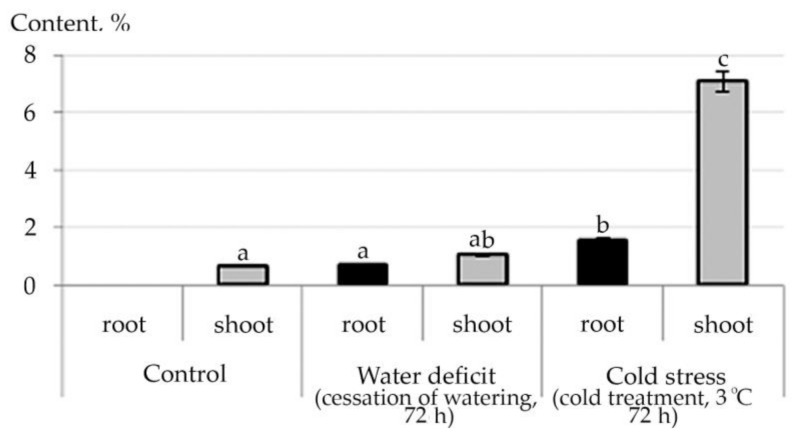
Change in concentration content of Diisooctyl phthalate (DIOP) in *R. semenowii* under stress conditions. Values presented are means (±SD). Different letters above the bars represent significant differences at *p* ≤ 0.05, *n* = 3 plants in each of 3 replicates for all treatments.

The content of esters of lower and middle carboxylic acids is presented in Supplementary Tables S1 and S2. An increase in the concentration of Diisooctyl Phthalate (DIOP) was noted under the action of the studied abiotic stressors, both in root and in shoot (Figure 12).

A significant increase in the content of Phosphoric acid, diethyloctyl ester was shown under conditions of osmotic stress, and Phosphoric acid, diethylnonyl ester was found under conditions of cold stress in root (Supplementary Tables S1 and S2).

**Figure 13:**
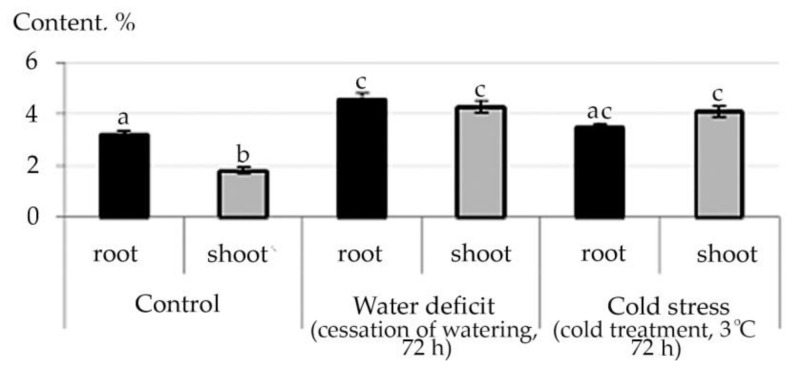
Change in content of Squalen in *R. semenowii* under stress conditions. Values presented are means (±SD). Different letters above the bars represent significant differences at *p* ≤ 0.05, *n* = 3 plants in each of 3 replicates for all treatments.

Abiotic stresses caused an increase in the content of diterpene (phytols) in shoot (Supplementary Tables S1 and S2) and triterpene (Squalen) hydrocarbons in both root and shoot (Figure 13).

## 3. Discussion

As a rule, the plant at different sensory levels perceives both water deficiency and cold. A decrease in biomass under water deficit is due to the loss of water by plant tissues and can serve as one of the indicators of water stress [34,35]. Generally, hypothermia also limits the growth and development of plants, although it does not always reduce the accumulation of biomass, preserving or increasing the water content of plant tissues. Nevertheless, it has such effects as a violation of the stability of proteins or protein complexes and a decrease in enzymatic activity [36]. Both water stress and cold lead to photoinhibition and disruption of photosynthesis, as well as significant membrane damage. The results of the study with water deficiency indicate damage to chlorophyll-bearing tissues, an increase in membrane permeability (the dye intensively penetrates into cells), a decrease in the content of free water in plant tissues, and a deterioration in the functioning of the conducting system in both root and shoot. In this case, water deficiency leads to a decrease in turgor, the development of plasmolysis and an increase in the concentration of cell juice and cytosol [37,38]. The high density of plant tissues of the leaf and the stomata immersed in the mesophyll are adaptive features that ensure a decrease in water loss during transpiration. Cold stress also causes damage to chlorophyll-bearing tissues, provoking numerous violations of the ultrastructure of cell membranes. The most common is an increase in the viscosity of their lipid part. The electron density of the cytoplasm decreases; structural changes occur (disintegration of granules, accumulation of lipid droplets, and the disappearance of starch grains). Therefore, membrane-bound processes such as photosynthesis and respiration are sensitive to cold stress [39]. In this way, the changes in the anatomical parameters of the leaf and stem of *R. semenowii* under stress conditions can also indicate damage to the photosynthetic apparatus.

A reduction in the values of the maximum quantum yield of photosystem II (PSII) is an indicator of photoinhibition and a decrease in the performance of PSII reaction centers [40]. At the same time, a decrease in the rate of noncyclic electron transport through PSII (ETR) indicates the activation of non-photochemical mechanisms of quenching [41], which points out a certain disruption in the functioning of the photosynthetic apparatus of *R. semenowii* under the created stress conditions. Any increase in Y (NPQ) under stress is an attempt to dissipate excess energy. Moreover, an increase in the quantum yield of uncontrolled heat dissipation and fluorescence Y (NO) means that the excess energy flows are out of control. High Y (NO) values at a relatively low Y (NPQ) in the studied plants indicate serious issues with the redistribution of excess light energy entering PSII [42,43]. This means it indicates possible damage to the photosynthetic apparatus of young plants of *R. semenowii*, caused by both water and cold stresses.

During their evolution, plants have adapted to drought conditions by accumulating SMs, however, increased SM accumulation is usually accompanied by reduced biomass [12]. Changes in the morphophysiological characteristics of plant organs growing under stress conditions are considered important acclimatization indicators [44]. However, the synthesis of SM under stress is also a significant component that is involved in defense reactions in response to biotic and abiotic stresses [45].

Oxidative stress is considered as one of the major causes of plant damage from abiotic stress, which is associated primarily with impaired electron transport in electron transport chains, caused by a change in the state of lipids.

A change in the parameters of the water balance or temperature causes a sharp increase in the generation of reactive oxygen species in the plant cell, and the cell needs antioxidant protection. The inhibition of free radicals in the plant is carried out by the antioxidant system. Due to the fact that SM has strong antioxidant properties, they may be associated with a mechanism for combating the harmful effects of reactive oxygen species (ROS), most of SM participating in maintaining redox balance by ROS scavenging (which also confers stress tolerance in plants) [7,13]. Antioxidants can be divided into two classes such as enzymatic antioxidants and nonenzymatic PSM [46,47], which we consider in this work.

The initial stage of autooxidation in membranes is inhibited by ubiquinones (tocopherol), polyphenols and superoxide dismutase. The ability to inhibit lipid peroxidation reactions is inherent only in the reduced forms of natural antioxidants and is associated with the presence of a hydroxyl group in the molecule. Natural antioxidants have a labile hydroxyl group and react relatively easily with hydrocarbon peroxide radicals [45].

For ubiquinones, the quinone form is the most stable. Tocopherols are found in lipids mainly in cyclic form which is both in the form of free tocopherol and in the form of its esters. In the body, the ester forms are easily hydrolyzed to free tocopherol with the help of enzymes. The increased content of γ-Tocopherol in shoot under stress conditions both water deficit and cold, confirms that tocopherols function as antioxidants in oxidative reactions. They form phenoxyl radicals by reacting with peroxide radicals, which are then converted into quinones, dimers, trimers. The study of lipid extracts from stress photodegradable cells demonstrates that enhanced autooxidation of vitamin E occurs with the production of 4, 8, 12, 16-tetramethylheptadecan-4-olide [48]. In our experiment, this phenomenon was found in shoot of *R. semenowii* against the background of stressful influences. Therefore, given the decrease in the photosynthetic ability of the plant when exposed to water deficit or low temperature, it is possibly also associated with photodegradation of cells.

Both the direction of the pharmacological action and its specificity are necessarily determined by glycosides, which are formed by sterols in reaction with carbohydrates, significant amounts of which have been identified in the ethanol extract. Phytosterols are bioactive compounds that are an important structural component of plant cell membranes in nature and play a vital role in the regulation of membrane fluidity and permeability. Moreover, phytosterols have a chemical structure similar to cholesterol obtained from mammalian cells. Among the various phytosterols, beta-sitosterol (SIT) and its stereoisomer gamma-sitosterol is the main compound found in abundance in plants. The primary precursor of sterol biosynthesis is cycloartenol, formed from squalene by cycloartenol synthase (CAS) [49]. Therefore, an increase in the level of SIT in root under stressful conditions both water deficit and cold stress, indirectly indicates its role in strengthening the membranes of plant cells.

The major building blocks of botanical membranes are phospholipids and glycolipids, both of which contain a glycerol core linked to two tails derived from fatty acids (FAs). As a result, FAs have a strong influence on the properties of membranes [50].

Both exposure to cold and water stress can cause disturbances in the conductivity of the biomembrane. It is proven by deactivation of proteins and ion leakage [51]. Reducing the fluidity of the cell membrane after exposure to stress is considered the first line of defense [52]. It has been shown in the literature that the fluidity of the plasma membrane correlates with the proportion of unsaturated fatty acids (UFAs) [53]. We experimentally revealed a decrease in the proportion of unsaturated FA in shoot both under water deficit and cold stress conditions. Furthermore, it has been shown that under stress with an increased content of UFAs in the lipids of the inner membranes of chloroplasts and mitochondria, the weakening of PSII photoinhibition may be associated [54,55]. Linoleic and linolenic fatty acids are essential and included in the composition of cell membranes, regulating their microviscosity, permeability, electrical properties, reducing excitability, forming the corresponding lipid environment of membrane proteins and enzymes [56]. In plants, fatty acids (FAs) can be present not only in a free state, but in the form of their methyl, ethyl, and other esters as well [57]. The nature of lipid components can vary greatly and include waxes, hydrocarbons (including squalene), sterol esters, aliphatic aldehydes, primary and secondary alcohols, 1,2-, 2,3- and α, ω-diols, ketones, β-diketones, triacylglycerols and numerous others. It was experimentally revealed that under stress conditions of both water deficit and cold, the content of FA in shoot esters significantly increases.

The interaction between plants and the environment is provided for the aerial organs by epicuticular waxes, which typically contain esters of long chain fatty alcohol esters with long chain fatty acids. However, little is known about the nature, biosynthesis and role of waxes at the root–rhizosphere interface. In this context, the observed changes in the content of Wax monoesters in the root of *R. semenowii* under abiotic stresses stimulated the interest. Li et al. [58] suggest a direct metabolic relationship between some root waxes and suberin. The direct physical connection between wax and suberin implies extracellular location under the primary cell wall, since suberin is deposited outside the plasma membrane. It is possible that most root waxes can be embedded deeper into the peridermal cell walls, where suberization occurs [58].

Chemicals from fatty acid metabolism can act as important chemical signals. Superoxide radical anion and hydrogen peroxide, which are produced under oxidative stress, can directly oxidize lipids or be converted to a hydroxyl radical via the Fenton and Haber-Weiss reactions [59]. The hydroxyl radical easily initiates the peroxidation of polyunsaturated, mainly linoleic and linolenic fatty acids. Spontaneous rearrangements of oxidized polyunsaturated fatty acids (PUFAs) lead to the formation of various phytoprostanes and aldehydes and other reactive electrophile species (RES), which are often toxic to plants [60]. Reactive oxygen species, especially singlet oxygen, formed in chloroplasts under stressful conditions, can also oxidize carotenoids, which also leads to the formation of such oxidized products as aldehydes, ketones, endoperoxides, and lactones [61]. As an example of this in our experiment is the detection of Benzeneacetaldehyde in shoot under cold stress as well as an increase in the concentration of 2-Hydroxy-gamma-butyrolactone in root under water deficit conditions and a decrease in it under conditions of cold stress with complete absence in stress conditions in shoot.

Perhaps, stress conditions that negatively influence the fragile and sensitive shoot are able to stimulate the internal solvation of the critical transition state by the neighboring hydroxyl group due to the binding and/or orientation of water molecules as a result of stress. The biological effects of linoleic and linolenic acids are realized at the cellular and organ levels. In response to abiotic stress in plants, lipases can be activated, which release unsaturated fatty acids and trigger the synthesis of a number of oxylipins with different functions [62]. Some of them have direct antimicrobial functions, while others are powerful regulators of defense mechanisms. Oxylipins are involved in plant acclimation to abiotic stresses as well. They are part of complex interactive networks of phytohormones, including salicylic acid, ethylene, auxin, brassinosteroids, gibberellic acid and abscisic acid, which control all aspects of plant growth and development and how plants adapt to their environment. These are signaling molecules formed from the group of polyunsaturated fatty acids, which are involved in the formation of the body’s responses to signals from the external environment [62].

Compared with other kinds of raw materials, the content of fatty acids in plants is low. Saturated and unsaturated fatty acids are part of the acyl lipids of plant tissue. Lipids, in turn, actively change metabolism and increase plant resistance, particularly to low temperatures [63,64]. Perhaps, it is the oxidative stress caused by the action of abiotic stressors that initiates the increase in the plant tissues of *R. semenowii* of such furan compounds as 2 (5H)-Furanone and Benzofuran. The appearance in the phytochemical spectrum of 2,5-Dimethyl-4-hydroxy-3 (2H)-furanone in root under cold stress also stimulated the interest.

It is noteworthy that the pyrrolidine ring is one of the most frequent heterocycles in the structure of medicines. This structural fragment is a part of many biologically active natural compounds (alkaloids nicotine, hygrin, the amino acid proline, etc.), among which there are osmolytes actively accumulated in plant tissues as a result of stress [65]. It is possible that the functions of osmolytes can be performed in this case by Methylglucoside (appearance in root under conditions of water deficiency).

In determining the directions of the potential pharmacological action of drugs based on *R. semenowii*, special attention should be paid to essential oils, which are based on various structures and in the presence of certain functional groups, terpenes, phenols, polyunsaturated carboxylic acids containing 1–4 double and triple bonds, ethers, alcohols [65]. Vegetable essential oils are intended to mediate the plant’s attitude to abiotic and biotic stress factors of various nature and to increase antioxidant activity [66,67,68]. In this context, free volatile substances are glycosylated, which are stored in the cell vacuoles and the internal swelling of the cells reduces the stress effect, particularly, from water deficiency [69]. This might be related to a decrease in leaf area [70].

Therefore, phenols exhibit antimicrobial and antioxidant (membrane stabilizing, cytoprotective) action. The antioxidant effect of phenols is responsible for stabilizing the cell membrane; phenols prevent mitochondrial autolysis. Moreover, they are involved in the suppression or blocking of free radicals, the most characteristic reaction of lipid peroxidation (LPO), and generally have a cytoprotective effect [71]. It is possible that the results of this experiment indicate precisely the antioxidant effect of phenols for plants under osmotic stress caused by water deficiency.

In the metabolism of higher plants, Benzoic acid (BA) influences their growth, anatomy, morphology, and stress resistance [72]. In plants, BA is a precursor of a wide range of primary and secondary metabolites, including various esters. Various modifications in BA molecules affect the volatility, permeability of substances in various cell compartments, their solubility and activity, and are crucial for their transport and functioning. Senaratna et al. [73] suggest that the structural part of benzoic acid is most likely the main functional molecular structure that makes plants resistant to stress. In this regard, the revealed changes in the concentrations of various BA esters in *R. semenowii* in shoot and root tissues under water deficit and cold stressful conditions arouse the interest for further research.

Unsaturated fatty acids such as palmitic acid are required to maintain a certain level of membrane fluidity. In the case of formic acid, in addition to acidic properties, it also exhibits some properties of aldehydes, notably, reducing properties. Unsaturated fatty acids are present in plant organisms in the form of esters. There is information about their connection with the acclimation of plants to low temperatures [74]. The data of our experiment also testify to their adaptive role to water deficit.

Esters of lower and medium carboxylic acids are constituents of the essential oils of many plants. The known phosphoric acid esters are extremely numerous. The majority of them play a central role in life processes and therefore they are of direct biological interest. In addition, there are crop improvers, plant growth regulators, ripening agents and others. Generally, in small quantities, they are involved in various processes taking place in a living organism and are aroma-forming components. We detected experimentally Diisooctylphthalate (DIOP) (diester) of phthalic acid (1,2-benzenedicarboxylic acid) which is the simplest representative of dibasic aromatic carboxylic acids. Commonly, the presence of phthalic acid is not typical of plant matter. In the case of detection of dialkyl phthalates in various objects and the interpretation of the data obtained is considered a serious problem [75]. However, it was noted that in response to stress induction by phytophages or phytopathogens, the content of diethyl phthalate and diisooctyl phthalate in plants could increase by more than five times [76]. For those in this experiment in conditions of both water deficiency and cold in shoot and in root, an incompletely understood stress response takes place.

Terpenes like other volatile components of green leaves (aldehydes, alcohols, and ethers) can serve as a signal of stress transmitted from plant to plant [77]. Diterpene hydrocarbons phytols are part of chlorophyll; therefore, there is no doubt about the relationship of their accumulation in the plant *R. semenowii* under stressful backgrounds, when the photosynthetic activity changes significantly. In this regard, the detected accumulation of phytols in shoot against the background of cold stress is of interest.

Squalen is a naturally occurring triterpene hydrocarbon. It belongs to the group of carotenoids. The accumulation of carotenoids in shoot is also closely related to stress acclimation processes. The decay products of hexoses serve as the basis for the synthesis of carotenoids and terpenes in a plant. In that case, carotenoids play an active part in the absorption of light energy shoot and its transfer to the reaction centers of the photosystem and serve as photoprotectors. Carotenoids protect the photosynthetic apparatus from photooxidizing damage by quenching the triplet of chlorophyll molecules [62]. Carotenoids are potent scavengers of reactive oxygen species that protect pigments and unsaturated fatty acids from lipids from oxidative damage [78]. They can react with free radicals by electron transfer, transfer of a hydrogen atom or its addition, as well as by modulating the physical properties of photosynthetic membranes with the participation of the xanthophyllic cycle in this process [79]. The positive effect of carotenoids on root growth was proven experimentally. Physical and physicochemical studies of the reactivity of carotenoids in redox processes are of priority today [80]. Therefore, the accumulation of Squalen in both shoot and root can be considered an adaptive stress response of the species *R. semenowii*.

Therefore, we can talk about the nonspecificity of many physiological and phytochemical reactions of plant tissues of *R. semenowii* to the effect of sudden cold or water stress. These reactions are expressed in a decrease in the level of photosynthetic activity, an increase in the content of such SM as γ-Tocopherol in shoot, SIT in root, a decrease in the proportion of unsaturated FA and an increase in the content of FA in shoot esters, in the accumulation of Squalen under stress in both shoot and root and others.

Nevertheless, specific adaptive mechanisms and nuances of reacting both shoot and root of *R. semenowii* to the action of each of the studied abiotic stresses were also noted. These are, for example, a change in the concentrations of various BA esters in the tissues of *R. semenowii*, the accumulation of phytols in shoot under cold stress conditions, as well as the pathways for the formation of such oxidized products as aldehydes, ketones and lactones and their derivatives, esters of lower and medium carboxylic acids, etc.

The variety of mechanisms of acclimation of *R. semenowii* plants to the abiotic stresses action by changing the SM content stimulates the interest for further experiments. In general, further research should be focused on the use of abiotic stressors for the targeted synthesis of bioactive SMs valuable for pharmaceutical use.

## 4. Materials and Methods

### 4.1. Plant Material and Growing Conditions

Plants *R. semenovii* grown in vegetation pots were studied. The plants had already lost their juvenile characteristics but had not entered the generative period of ontogenesis yet during the immature period of development, when an intensive growth of the shoot was observed.

Plants at the time of the experiment were divided into three groups: (1) a control group were grown under 26 ± 3 °C at day and 20 ± 3 °C at night, with average air humidity 37% and optimal irrigation (up to 60% of full moisture capacity); (2) a group subjected to sudden cold treatment +3 °C in refrigerator cabinet with lighting (“Polair”, Moscow, Pussia) under circadian illumination (using commercial fluorescent white light tubes): 16 h light/8 h darkness regime [200 µmol m^−2^ s^−1^ PAR, light metre LI-205 (Li-Cor, Lincoln, NE, USA)]) and (3) a group subjected to water deficiency (cessation of watering). The duration of the stress exposure was 72 h.

The calculation of the soil moisture was carried out according to the formula: W= (a x 100): b (%), where (W)—the soil moisture, in % of the dry soil mass; (a)—the mass of water in the soil sample, g; (b)—dry soil mass, g.

Growth parameters were determined by measurements before and on the third day after the onset of stress exposure [81]. The water content (WC) in plant tissues was calculated using the formula:

WC = ((a − б)/a) × 100%,(1)

where a is the initial mass, mg; b is the mass after drying at 105 °C, mg.

### 4.2. Photosynthetic Activity Determination

Photosynthetic activity parameters were estimated by determination of fluorescence levels. Rapid light curves (RLCs) were recorded using Junior-PAM (“Heinz WalzGmbH”, Effeltrich, Germany) under actinic illumination of 450 nm. The RLC for each sample was recorded after quasi-darkness to assess the effect of actinic light absence, while complete darkness is difficult to achieve under field conditions [82]. For each measurement the fluorometer provided eight saturation light pulses of 10,000 µmol/m^2^s every 20 s, while actinic light increased from 0 to 625 µmol/m^2^s gradually. For comparison, the data obtained from the last pulse of the light curve were taken [83]. The following parameters were calculated using WinControl-3.29 (Walz, Effeltrich, Germany) software: Fv/Fm: maximum quantum yield of PSII photochemistry; Y(II): effective photochemical quantum yield of PSII; Y(NPQ): quantum yield of non-photochemical energy conversion in PSII due to downregulation of the light-harvesting function; Y(NO): quantum yield of non-photochemical energy conversion in PSII that caused by downregulation of the light-harvesting function; PSII relative electron transport (ETR). In the experiment, each time the region of the middle third of the active leaf was selected. All measurements were performed on a sunny day from 09:00 to 11:00 a.m.

### 4.3. Analysis of Changes in the Elements of the Anatomical Structure

Fixation of roots was performed in 70% ethanol and preservative fluid was a Strasburger-Flemming’s mixture: 96% ethanol:glycerol:water in ratio of 1:1:1 [84]. The material was infused for a 24 h. Anatomical specimens were prepared with a microtome MZP-01 (“Technom”, Ekaterinburg, Russia) with a freezing unit OL-ZSO 30 (“Inmedprom”, Yaroslavl, Russia). The thickness of anatomical sections varied between 10 and 15 microns. Sudan IV-stained sections were placed on a glass slide in a drop of pure glycerin and covered with a cover slip to obtain a temporary preparation. Micrographs of anatomic sections were made on a microscope with Micro Opix MX 700 (T) (West Medica, Brown Boveri-Straße 6, B17-1 2351 Wiener Neudorf, Austria), CAM V1200C HD-camera (West Medica, Brown Boveri-Straße 6, B17-1 2351 Wiener Neudorf, Austria). All anatomical data were obtained in 3–5 replicates (5 plants in each) with a 40× objective.

### 4.4. Determination of Organic Compounds in Extracts

Analysis methods: Gas chromatography with mass spectrometric detection (Agilent 6890N/5973N, Santa Clara, CA, USA). Sample volume 1.0 μL, sample injection temperature 260 °C, without flow division. Separation was carried out using a chromatographic capillary column DB-35MS with a length of 30 m, an inner diameter of 0.25 mm, and a film thickness of 0.25 μm at a constant carrier gas (helium) velocity of 1 mL/min. The chromatographic temperature was programmed from 40 (exposure 0 min) to 150 °C with a heating rate of 10 °C/min (exposure 0 min) and up to 300 °C with a heating rate of 5 °C/min (exposure 10 min). Detection was carried out in the SCANm/z 34-850 mode. AgilentMSDChemStation software (version 1701EA) (Santa Clara, CA, USA) was used to control the gas chromatography system, register and process the obtained results and data. Data processing included determination of retention times, peak areas, as well as processing, spectral information obtained using a mass spectrometric detector. The Wiley 7th edition and NIST’02 libraries were used to decode the obtained mass spectra (the total number of spectra in the libraries is more than 550 thousand).

All experiments were done in three replicates. The processing of data and graphing was performed using Microsoft Excel (Microsoft Corp., Redmond, Washington, DC, USA). Atypical values were excluded from the data based on t-tests, the standard error of the average sample was calculated. Differences were considered significant at *p* < 0.05.

## 5. Conclusions

The results of this study demonstrated that under water deficit and cold stress conditions, morphophysiological responses and elements of anatomical structure of organs of *R. semenovii* changed at some degrees.

For the first time, a detailed study of the chemical composition of the ethanol extract of root and shoot of *R. semenovii* under stress was carried out using gas chromatography–mass spectrometry, which made it possible to state that the antioxidant system in plant tissues is multicomponent and includes SM. All components are in functional interaction and are due to the body’s adaptive stress responses.

The results obtained for SM, which are medicinal biologically active substances, are useful both for understanding the mechanisms of protection against adverse conditions and for approaches to the targeted synthesis of secondary metabolites valuable for pharmaceutical applications.

## Data Availability

Not applicable.

## Supplementary Materials

The following are available online at https://www.mdpi.com/article/10.3390/plants10061196/s1, Table S1: Change in content of SM in shoot under stress conditions, Table S2: Change in content of SM in root under stress conditions.
