# Supplementary material for: The Influence of Abiotic Stress Factors on the Morphophysiological and Phytochemical Aspects of the Acclimation of the Plant Rhodiola semenowii Boriss
Source: Plants (Basel). 2021 Jun 11;10(6):1196. doi: 10.3390/plants10061196 (PMC8231149; doi:10.3390/plants10061196)
Supplement: Supplementary file 1 [file plants-10-01196-s001.zip › plants-1242788-supplementary.pdf]

Table S1. Change in content of SM in shoot under stress conditions.

	Control			Water deficit			Cold-stress		
	Content, %	Reten-tion time, min	Iden-tifica-tion proba-bility, %	Content, %	Reten-tion time, min	Iden-tifica-tion proba-bility, %	Content, %	Reten tion time, min	Iden-tifica-tion proba-bility, %
ubiquinones									
4,8,12,16-Tetramethylheptadecan-4-olide	0.88	39.2	69	1.35	39.2	77	1.79	39.2	65
γ-Tocopherol	0.77	49.9	65	1.97	49.9	76	1.39	49.9	63
phytosterols									
Lup-20(29)-en-3-one	8.57	46.8	74	-	-	-	-	-	-
fatty acids									
17-Octadecynoic acid	7.97	28.0	68	-	-	-	-	-	-
fatty acid esters									
Hexadecanoic acid, ethyl ester (palmitic acid ester)	2.41	31.5	72	3.08	31.5	76	-	-	-
Ethyl Oleate (oleic acid ester)	-	-	-	-	-	-	1.38	35.0	70
9,12-Octadecadienoic acid, ethyl ester (linoleic acid ester)	2.63	35.2	84	2.94	35.2	82	6.10	35.2	81
Ethyl 9,12,15-octadecatrienoate (Ethyl 9α-linolenate, linolenic acid ethyl ester)	-	-	-	2.73	35.5	87	3.97	35.5	84
Hexadecanoic acid, 1-(hydroxymethyl)-1,2-ethanediyl ester (palmitic acid ester)	0.99	38.1	63	0.47	38.1	62	1.49	38.1	66
aldehydes, glycosides, alcohols, hydrocarbons, amino acids and their derivatives									
1-Octen-3-ol	0.65	11.2	71	0.65	11.2	71	-	-	-
Benzeneacetaldehyde	-	-	-	-	-	-	9.64	14.7	92
Cyclopropyl carbinol	3.32	17.1	76				0.96	17.1	60
2-Propanol,1-chloro-, phosphate	-	-	-	-	-	-	2.64	30.7	71
cyclic five-membered ketones, lactones and their derivatives									
4-Cyclopentene-1,3-dione	2.17	11.0	87	2.44	11.0	88	1.86	11.0	78
2-Cyclopenten-1-one, 2-hydroxy-	4.99	11.8	85	-	-	-	2.43	11.8	65
1,2-Cyclopentanedione	-	-	-	3.98	11.8	88	-	-	-
Butyrolactone	-	-	-	1.31	12.7	81	1.91	12.7	69
1,2-Cyclopentanedione, 3-methyl-	1.44	14.2	85	-	-	-	-	-	-
2-Hydroxy-gamma-butyrolactone	7.25	14.7	80	-	-	-	-	-	-
2-Cyclopenten-1-one, 2-hydroxy-3-methyl-	-	-	-	1.60	14.2	85	-	-	-
ketones									
2-Propanone, 1-(acetyloxy)-	1.35	10.5	91	2.18	10.5	89	1.39	10.5	70
1-Phenyl-2-butanone	-	-	-	9.33	14.7	66	-	-	-

(Estragol,Benzylacetone, Butyrophenone)									
Furan and Pyran derivatives									
2(5H)-Furanone	1.57	12.8	80	1.22	12.8	77	0.51	12.8	62
2(5H)-Furanone, 3-methyl-	3.23	14.0	88	3.11	14.0	88	1.79	14.0	70
β-D-Glucopyranose, 1,6-anhydro-	2.10	25.8	63	-	-	-	-	-	-
Ethyl α-d-glucopyranoside	17.71	27.8	83	15.69	27.8	82	-	-	-
phenols									
Phenol,2,6-dimethoxy-	0.78	21.7	76	-	-	-	-	-	-
Benzoic acid derivatives (esters)									
Benzoic acid, heptyl ester	0.69	29.9	66	0.55	29.9	63	-	-	-
Benzoic acid, tridecyl ester	-	-	-	0.68	30.4	65	1.37	30.4	62
Benzoic acid, pentadecyl ester	0.59	30.3	60	-	-	-	-	-	-
saturated monobasic acids esters									
Formic acid, 2,6-dimethoxyphenyl ester	-	-	-	0.91	21.7	72	-	-	-
Hexadecanoic acid, ethyl ester	2.41	31.5	72	3.08	31.5	76	-	-	-
Hexadecanoic acid, 1-(hydroxymethyl)-1,2-ethanediyl ester	0.47	38.1	62	0.99	38.1	63	1.49	38.1	66
phthalic acid esters									
Diisooctyl phthalate (DIOP)	0.67	42.6	61	1.05	42.6	71	7.08	42.6	91
Phthalic acid, butyl hept-3-yl ester	-	-	-	-	-	-	0.66	33.5	75
phosphoric acid esters									
Phosphoric acid, diethyl octyl ester	1.49	24.0	72	2.13	24.0	71	-	-	-
Phosphoric acid, diethyl nonyl ester	-	-	-	-	-	-	2.08	24.0	69
di- and triterpenes									
3,7,11,15-Tetramethyl-2-hexadecen-1-ol	7.32	27.4	88	7.25	27.4	88	20.14	27.4	90
Phytol	5.51	33.7	91	5.89	33.7	90	17.92	33.7	91
Squalen	1.81	45.2	80	4.29	45.2	84	4.08	45.2	83

Table S2. Change in content of SM in root under stress conditions.

	Control			Water deficit			Cold-stress		
	Content, %	Reten-tion time, min	Iden-tifica-tion proba-bility, %	Content, %	Reten-tion time, min	Iden-tifica-tion proba-bility, %	Content, %	Reten tion time, min	Iden-tifica-tion proba-bility, %
ubiquinones									
γ-Tocopherol	1.84	49.9	73	1.55	49.9	75	0.98	49.9	60
phytosterols									
β-Sitosterol	2.64	48.6	60	-	-	-	3.99	48.6	66
γ-Sitosterol	-	-	-	2.32	48.6	63	3.12	48.6	64
fatty acids									
Propanoic acid, 3-(acetylthio)-2-methyl-	-	-	-	0.90	18.2	66	-	-	-
fatty acid esters									
Hexadecanoic acid, ethyl ester (palmitic acid ester)	9.55	31.5	88	8.42	31.5	88	8.63	31.5	78
Ethyl Oleate (oleic acid ester)	0.99	35.0	65	1.15	35.0	70	0.95	35.0	66
9,12-Octadecadienoic acid, ethyl ester (linoleic acid ester)	12.86	35.2	90	12.09	35.2	89	12.27	35.2	89
Ethyl 9,12,15-octadecatrienoate (Ethyl 9α-linolenate, linolenic acid ethyl ester)	5.26	35.5	88	4.15	35.5	88	3.82	35.5	86
Hexadecanoic acid, 1-(hydroxymethyl)-1,2-ethanediyl ester (palmitic acid ester)	-	-	-	0.90	38.1	61	-	-	-
aldehydes, glycosides, alcohols, hydrocarbons, amino acids and their derivatives									
1,2-Ethanediol, diacetate	-	-	-	-	-	-	1.60	10.5	85
3-Octanethiol, thiolacetate	0.72	13.5	60	-	-	-			
Cyclopropyl carbinol	4.01	17.1	78	5.50	17.1	77	-	-	-
l-Alanine, N-(2-furoyl)-, propyl ester (pharinomalein, natural maleimide)	-	-	-	1.20	20.1	66	-	-	-
1-Docosanol, acetate	4.82	42.0	77	-	-	-	6.45	42.0	72
Tetratetracontan	-	-	-	-	-	-	2.52	42.1	69
Tetracosyl acetate (Wax monoesters)	9.40	44.9	88	5.97	44.9	85	15.11	44.9	90
Octacosyl acetate	2.15	47.7	65	-	-	-	3.08	47.7	68
cyclic five-membered ketones, lactones and their derivatives									
4-Cyclopentene-1,3-dione	1.98	11.0	84	2.59	11.0	86	2.34	11.0	85
2-Cyclopenten-1-one, 2-hydroxy-	3.80	11.8	87	4.81	11.8	88	-	-	-
1,2-Cyclopentanedione	-	-	-	-	-	-	3.53	11.8	74
Butyrolactone	1.49	12.7	75	2.02	12.7	74	1.65	12.7	64

1,2-Cyclopentanedione, 3-methyl-	0.81	14.2	88	1.27	14.2	89	0.70	14.2	80
2-Hydroxy-gamma-butyrolactone	4.27	14.7	88	8.39	14.7	85	4.10	14.7	86
ketones									
2-Propanone, 1-(acetyloxy)-	1.34	10.5	85	1.89	10.5	92	-	-	-
5,9-Dodecadien-2-one, 6,10-dimethyl-, (E,E))- (Ethyl geranyl acetone)	-	-	-	1.91	19.7	63	1.11	19.7	65
Furan and Pyran derivatives									
2(5H)-Furanone	1.99	12.8	79	3.38	12.8	89	2.26	12.8	77
2(5H)-Furanone, 3-methyl-	-	-	-	0.57	14.0	77	-	-	-
2,5-Dimethyl-4-hydroxy-3(2H)-furanone	-	-	-	-	-	-	5.64	14.8	68
2(3H)-Furanone, 5-acetyldihydro-	-	-	-	0.90	18.2	66	-	-	-
Benzofuran, 2,3-dihydro-	2.03	18.4	75	2.55	18.4	78	-	-	-
4H-Pyran-4-one, 2,3-dihydro-3,5-dihydroxy-6-methyl (3-Hydroxy-2,3-dihydromaltol)	1.22	16.9	68	1.18	16.9	71	1.27	16.9	63
α-D-Glucopyranose, 4-O-β-D-galactopyranosyl (cellobiose)	10.88	36.2	69	-	-	-	-	-	-
α-D-Glucopyranoside, methyl (Methyl glucoside)	-	-	-	12.55	36.2	68	-	-	-
phenols									
Phenol	-	-	-	0.61	12.6	76	-	-	-
Benzoic acid derivatives (esters)									
Benzoic acid, heptyl ester	0.70	29.9	66	-	-	-	1.14	29.9	67
Benzoic acid, tridecyl ester	0.85	28.8	62	0.28	28.8	63	-	-	-
Benzoic acid, tetradecyl ester				0.61	30.0	69			
Benzoic acid, pentadecyl ester	0.93	30.4	67	1.12	30.4	70	1.15	30.4	68
saturated monobasic acids esters									
Hexadecanoic acid, 1-(hydroxymethyl)-1,2-ethanediyl ester	-	-	-	0.90	38.1	61	-	-	-
Hexadecanoicacid, ethyl ester	9.55	31.5	88	8.42	31.5	88	8.63	31.5	88
phthalic acid esters									
Diisooctyl phthalate (DIOP)	-	-	-	0.70	42.6	61	1.55	42.6	88
Phthalic acid, di(2-propylpentyl) ester	0.95	42.6	64	-	-	-	-	-	-
di- and triterpenes									

Squalen	3.20	45.2	86	4.57	45.2	90	3.45	45.2	81
				oxybutyrates					
2-Hydroxy-gamma-butyrolactone	4.27	14.7	88	8.39	14.7	85	4.10	14.7	86
